# Surgical decision-making for ossification of the posterior longitudinal ligament versus other types of degenerative cervical myelopathy: anterior versus posterior approaches

**DOI:** 10.1186/s12891-020-03830-0

**Published:** 2020-12-08

**Authors:** Suzanna Sum Sum Kwok, Jason Pui Yin Cheung

**Affiliations:** Department of Orthopaedics and Traumatology, The University of Hong Kong, Professorial Block, 5th Floor, 102 Pokfulam Road, Pokfulam, Hong Kong, SAR China

**Keywords:** Cervical myelopathy, Ossification of the posterior longitudinal ligament, OPLL, Cervical spine, Degenerative cervical myelopathy, DCM, Cervical spondylotic myelopathy, CSM

## Abstract

**Background:**

The debate between anterior or posterior approach for pathologies such as cervical spondylotic myelopathy (CSM) and ossification of the posterior longitudinal ligament (OPLL) have drawn heated debate but are still inconclusive.

**Main body of the abstract:**

A narrative review was performed specifically to study the differences pertaining to OPLL and other causes of degenerative cervical myelopathy (DCM). Current evidence suggests that anterior approach is preferred for K-line (−) OPLL, K-line (+) with canal occupying ratio > 60% and DCM with pre-existing cervical kyphosis. Posterior approach is preferred for K-line (+) OPLL with canal-occupying ratio < 50–60%, and multi-level CSM. No particular advantage for either approach was observed for DCM in a lordotic cervical spine. Anterior approach is generally associated with more complications and thus needs to be weighed carefully during decision-making. The evidence is not convincing for comparing single versus multi-level involvement, and the role of patients' co-morbidity status, pre-existing osteoporosis and co-existent spinal pathologies in influencing patient outcome and surgical options. This should be a platform for future research directives.

**Conclusion:**

From this review, evidence is still inconclusive but there are some factors to consider, and DCM and OPLL should be considered separately for decision-making. Anterior approach is considered for pre-existing cervical kyphosis in DCM, for K-line (−) regardless of canal-occupying ratio, and K-line (+) and canal-occupying ratio > 60% for OPLL patients. Posterior approach is considered for patients with multi-level pathology for DCM, and K-line (+) and canal-occupying ratio < 50–60% for OPLL.

## Introduction

Cervical myelopathy is a dysfunction of the spinal cord and is often caused by a narrowing of the cervical spinal canal. This narrowing may be congenital or acquired or both [1]. In those with congenital narrowing, a minor degree of additional pathology such as disc protrusion may already lead to symptoms [2]. Prolonged compression of the spinal cord eventually leads to grey matter atrophy and cell body degeneration which results in neurological symptoms [1]. Cervical spondylotic myelopathy (CSM) is the most common cause of spinal cord dysfunction which occurs in the elderly [3, 4] and most commonly affects C5–6, followed by C6–7 and C4–5 [3]. Ossification of the posterior longitudinal ligament (OPLL) is also an important pathology. These ossification disorders are more common in Asians and may progress more aggressively [5]. These along with degenerative disc disease constitutes various non-traumatic and degenerative forms of cervical myelopathy and are commonly recognized under the umbrella term of degenerative cervical myelopathy (DCM) [6].

Patients with cervical myelopathy presents with weakness in the extremities, gait imbalance, abnormal reflexes, and clumsiness [7]. Clumsiness and sensory deficits may lead to functional difficulties including buttoning, picking up small objects and using chopsticks. Its natural history is usually progressive resulting in worsening disability and progressive limitation in function [8]. It is believed that 75% of cases progress in a step-wise manner, 20% deteriorate slowly and 5% have rapid onset of symptoms [9], and thus the majority will need surgical treatment [8].

Surgical decompression may be achieved via anterior or posterior approaches and is unclear when either method is more suitable or preferred. There has long been debate on the best approach for cervical myelopathy especially for multi-level compression. We believe that the entity of OPLL may be different from the rest of pathologies within DCM. In this narrative review, we will highlight the differences in the techniques currently used and their respective advantages and disadvantages with respect to OPLL and other causes of DCM separately as they are different pathologies, and to suggest current recommendations and insights. We included only articles that specifically differentiated OPLL and other causes of DCM like CSM in this review.

## Anterior approach

**Fig. 1:**
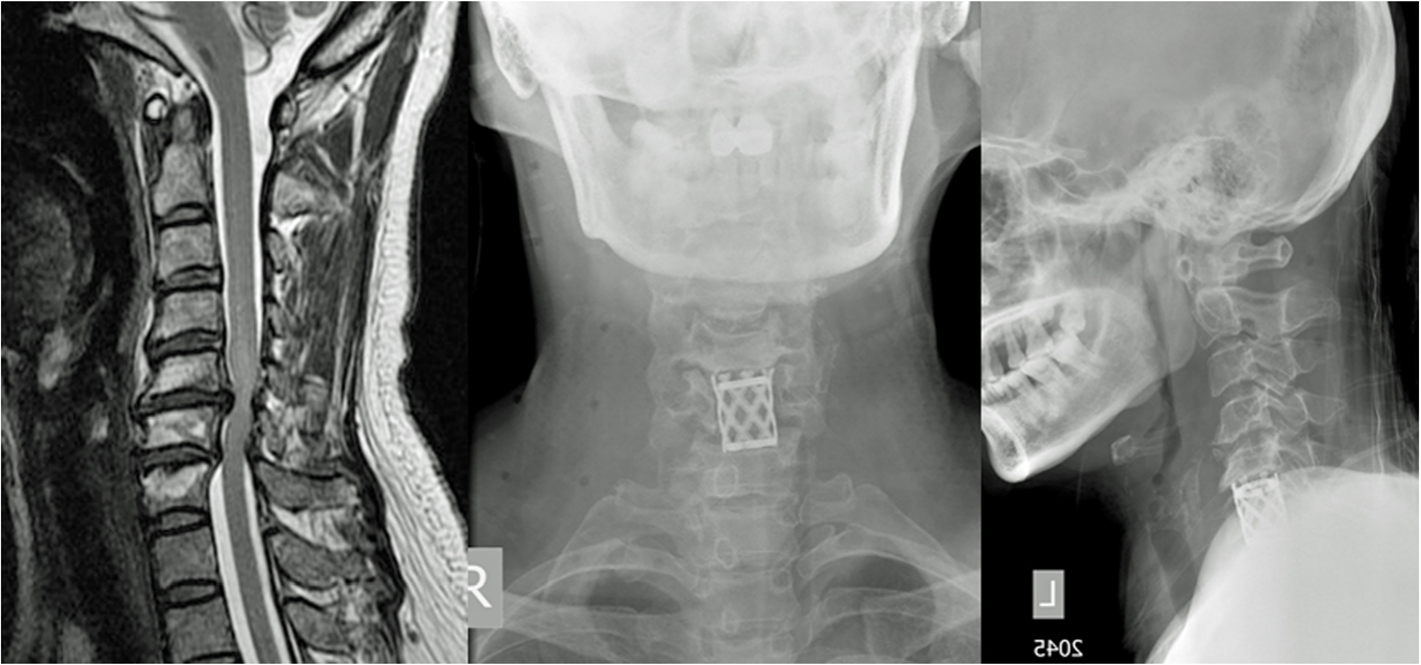
T2 weighted MRI image showing a patient with cervical spondylotic myelopathy involving C5-C6 and C6-C7 levels (left) who underwent an anterior cervical corpectomy and fusion from C5-C7. Post-operative AP (middle) and lateral (right) x-ray images show a cage with bone graft stabilizing the C5-C7 segments

The anterior approach entails removal of the disc or part of the vertebral body [3]. Direct removal of any disc herniation or osteophyte is possible along with the posterior longitudinal ligament [3]. After decompression however, fusion is usually necessary to restore spinal mechanical stability and integrity [3] and is achieved through the use of tricortical bone autograft [10] and/or implants like mesh cages and plates. Instrumentation may aid alignment correction and fusion of the segments [3]. These procedures are termed anterior cervical discectomy with fusion (ACDF) for disc removal or anterior cervical corpectomy with fusion (ACCF) for vertebral body excision (Fig. 1). ACDF is generally preferred for a patient with pre-existing cervical kyphosis as restoration of anterior column height may help restore normal lordotic curvature and prevents iatrogenic kyphosis due to posterior implant failure [3].

Generally speaking, anterior decompression with fusion and instrumentation has been deemed suitable for cervical myelopathy involving 1–2 levels with low incidence of complications [11, 12]. However, problems arise when more than two levels are involved and the anterior approach is associated with higher rates and unpredictable complications [13, 14]. Complications include implant failure, non-union, and graft dislodgment which occur at an unacceptable incidence of 17–45% [15, 16] according to different studies. Other typical complications such as dysphagia and hoarseness due to postoperative retropharyngeal oedema or recurrent laryngeal nerve injury are a concern for the anterior approach (Table 1) [17]. Reported rates of implant complications are 33.3% of cases, hoarseness and dysphagia in 28.2% of cases of anterior OPLL surgery [18]. There is also greater risk of perioperative complications such as greater chance of intra-operative haemorrhage [3]. Anterior approach also requires longer operative time [3] due to the high technical demand (Table 1). In addition, the anterior approach may be more challenging in osteoporosis. Osteoporotic bone poses greater surgical challenge due to poor bone and tissue quality which have shown greater risk of subsidence of strut grafts and higher failure rate for anterior plate placement or instrumentation [19]. Osteoporosis is an important factor to be taken into consideration as the majority who need surgery are elderly.

**Table 1:** General comparison between the two approaches

	Pros	Cons
**Anterior approach**	Better for those with pre-existing cervical kyphosis More direct approach to pathologies such as disc herniation and OPLL	**Intra-operative:** Longer operating time Higher intra-operative haemorrhage **Post-operative complications:** Longer hospitalisation Dysphagia and hoarseness of voice Higher graft related complications
**Posterior approach**	Simpler and extensile procedure Shorter operating times Faster recovery Virtually no chance of damaging vital structures in the neck	**Post-operative complications** More post-operative neck pain Higher intra-op kyphosis C5 palsy Hinge fracture Spring-back closure

*OPLL* Ossification of the posterior longitudinal ligament

Within each approach, there may be significant differences in outcome. A meta-analysis looked at 8 studies which compared ACDF with ACCF and found ACDF to be associated with better surgical and radiological outcomes compared to ACCF [20]. There were no significant differences in preoperative and final follow-up Japanese Orthopaedic Association (JOA) scores between ACCF and ACDF [20]. Radiological assessment was done by assessing the angle of C2-C7 pre- and post-operatively. The post-operative angle of C2-C7 with ACDF (20.3°) was better than ACCF (14.3°) with a standardized mean difference of 4.76 (*P* < 0.001) [20].

## Posterior approach

Posterior approach is preferred in the treatment of multi-level cervical myelopathy [1, 3] and is also necessary for excision of pathologies at the posterior part of the spinal canal such as ossified ligamentum flavum. It is an extensile approach with easy extension of the exposure to the occipital-cervical and thoracic regions. Traditionally, laminectomy was the gold-standard [3] but has been largely replaced by laminoplasty, especially in Asia, due to improved clinical outcomes [21] and less complications such as segmental instability, post-laminectomy kyphosis, perineural scar formation, loss of cervical motion, and delayed neurologic deterioration [22]. Cervical laminoplasty is an indirect method to decompress the spinal cord by widening the narrowed spinal canal while preserving the posterior anatomical structures as much as possible [1].

The two most commonly utilized methods of laminoplasty are the double-door laminoplasty where osteotomies are made at the central spinous process and the open-door laminoplasty where the osteotomy is done at the lamina-facet junction on one side [1]. Laminectomy involves resection of the spinous process along with the dorsal laminae to expose the ligamentum flavum for decompression and thus results in loss of the posterior soft tissue construct [23]. Laminectomy alone is mostly obsolete and laminectomy and fusion is commonly adopted in Europe and North America with good surgical outcome and quality of life [24]. The posterior approach is commonly used by most surgeons as it is a relatively simple approach with no vital structures at risk of injury [25, 26]. However, since the posterior approach involves muscle incision and strong retraction of the paravertebral muscles [27], the incidence of wound infection and other wound complications is higher with more post-operative neck pain due to soft tissue dissection and retraction (Table 1) [3, 28]. For open-door laminoplasty, another complication includes hinge fracture [29] but has been shown to reunify within 2 years after surgery without adverse effects in 90% of cases [30]. Spring-back closure is also an important complication of laminoplasty which has been shown to occur at a rate of 40% [31] but has only been found in those with suture fixation not with modern miniplate fixation [32]. For the posterior approach, C5 palsy is an important complication which occurs at an incidence rate of 1.4–23% resulting in weakness of the deltoid and biceps brachii [33] and in cases of more severe weakness the damage may be irreversible [34]. Risk factors of C5 palsy include older age, male, OPLL, preoperative foraminal stenosis, pre-existing deltoid weakness, and laminectomy and fusion [35, 36].

It is important to note that within each approach, there are variations in technique. This should be taken into consideration when planning for surgery. The current literature lacks an overall systematic approach for comparing different anterior and posterior options due to these variabilities. The following is a review of comparative literature that is specific to OPLL and other causes of DCM.

## Anterior vs posterior approach

### Degenerative cervical myelopathy (DCM)

#### Clinical outcomes

Audat et al [7] showed that there were no significant differences in the clinical and radiological outcome between anterior surgery and laminectomy with fusion used in the surgical treatment of DCM. The mean values of the Neck Disability Index (NDI) demonstrated better statistical results (*p* < 0.05) at all follow-up periods for the anterior approach versus the posterior approach [7]. The authors ultimately concluded that the anterior approach appeared to be superior based on the clinical outcomes (Table 2) [7]. Another prospective observational study looked at 278 subjects with the surgical option based on the patient’s age, level of involvement, primary site of compression and nature of the pathology in deciding the surgical treatment [37] (Table 2). Patients with more focal pathology underwent anterior surgery and those with multi-level cervical involvement were usually treated by posterior techniques. No significant differences were observed in the improvement of NDI scores between groups [37] A non-randomised study which looked into 75 patients with CSM stratified the outcomes by severity and recovery rate by the Japanese Orthopaedic Association (JOA) score [8]. In patients with mild disability (pre-op JOA score ≥ 14), their overall recovery rate was 100% while the moderate disability group (pre-op JOA score 10–13) had an overall recovery rate of 77.8% and the severe disability group (pre-op JOA score ≤ 9) had an overall recovery rate of 63.7% (Table 2) [8]. The recovery rates were otherwise comparable regardless of the approach within the same category of severity at presentation. Hence, this appears to be the main determinant of functional outcome (Table 2) [8].

**Table 2:** Comparisons for DCM

Reference	Type of study	No. of patients	Parameters measured	Key findings
Audat et al. 2018 (ref # [7])	Retrospective clinical study	287	Pre-operative and post-operative mean ± standard deviation for NDI	Anterior approach appeared to be superior based on the clinical outcomes
Zaveri et al. 2019 (ref # [8])	Non-randomised clinical study	75	Recovery rates of mild, moderate and severe CSM based on mJOA scores	Patient outcome mainly determined by clinical severity on presentation Recovery rates were comparable regardless of the approach within the same category of severity at presentation
Fehlings et al. 2013 (ref # [37])	Prospective observational study	278	NDI pre and post-operatively at 12 months	No significant differences between anterior vs posterior approach for NDI improvement
Luo et al. 2015 (ref # [38])	Meta-analysis and systematic review	467	Pre-operative and post-operative JOA scores recovery rate	No statistical difference in recovery rate between anterior and posterior approaches
Liu et al. 2011 (ref # [4]0)	Non-randomized controlled trial	52	JOA scores, recovery rate, range of motion	No differences between ACDF vs laminoplasty for JOA score and recovery rate Range of motion reduced in ACDF vs laminoplasty
Xu et al. (ref # [39])	Meta-analysis	379	JOA score, recovery rate	No differences between ACDF and laminoplasty

*ACDF* Anterior cervical discectomy and fusion, *CSM* Cervical spondylotic myelopathy, *DCM* Degenerative cervical myelopathy, *mJOA* Modified Japanese Orthopaedic Association, *NDI* Neck disability index

.A study compared the posterior approach with single-staged combined anterior and posterior approach decompression for those with multi-level CSM and concluded that neurological outcomes for both approaches were similar with no significant differences in the JOA score and visual analogue scale [40] (Table 2). Luo et al [38] conducted a meta-analysis showing that the postoperative JOA score was significantly higher in the anterior surgery group (JOA 13.7) compared with the posterior surgery group (JOA 13.0)(*P* < 0.05) albeit not reaching clinical significance (Table 2). The preoperative JOA scores of the anterior (JOA 8.96) and posterior (JOA 8.98) approaches showed no statistical differences [38]. Interestingly, there was no statistical difference in recovery rate between the anterior (56.3%) and posterior (53.8%) approaches (*P* > 0.05) [38]. Ultimately the authors could not come to a conclusion to which approach is most effective for multi-level CSM. However, given that the long term outcome is similar, the posterior approach seems more advantageous for multi-level CSM since it is safer with less complications.

A non-randomised randomized controlled trial comparing ACDF (JOA 13.2) vs laminoplasty (JOA 13.67) in 52 patients with multi-level CSM demonstrated that both approaches showed significant improvement in JOA score (*P* < 0.001) (Table 2). The recovery rates for ACDF was 59.79% versus 59.54% for laminoplasty (*P* > 0.05) [41]. They also compared the radiological outcome and ACDF resulted in reduced vertebral range of motion of 29.45% compared to 11.39% for laminoplasty (*P* < 0.05) [41]. Another meta-analysis compared ACDF vs laminoplasty in multi-level CSM concluded that ACDF (59.3%) and laminoplasty (66.8%) had no significant difference in JOA score recovery rate [39].

#### Complications

One study concluded that ACDF had similar complication rates of 16 and 11% respectively which included dysphagia, C5 radiculopathy, and axial neck pain [42]. Another study focusing on multi-level CSM showed the anterior group had a complication rate of 20.7% compared to 15.5% for the posterior approach (*P* = 0.009) [38]. The anterior approach has a reoperation rate of 4.2% compared to 0.2% for the posterior approach for multi-level CSM (*P* < 0.001) [38]. Specific complications to ACDF included late deterioration, screw loosening, pseudoarthrosis, temporary odynophagia and dysphonia. For posterior surgery, C5 palsy and axial neck pain are most prevalent (*P* < 0.05) [41]. ACDF also resulted in faster decline in ROM of the cervical spine than laminoplasty [41].

Studies also showed no significant difference in terms of duration of hospital stay. One study suggested 7 days for the anterior approach and 5 days for the posterior approach [38, 42]. Another study focusing on multi-level CSM showed that the anterior approach required shorter hospital stay of around 3.77 days and posterior approach required a hospital stay of around 4.13 days (*P* < 0.001) [38]. As for operating times, the anterior approach showed significantly longer operating time with the anterior approach (222.6 min) compared to the posterior approach (159.5 min)(*P* < 0.001) [38]. The anterior approach also has a blood loss of about 538 ml and the posterior approach has a blood loss of 454 ml (*P* < 0.05) [38, 42]. Another study focusing on multi-level CSM showed that ACDF resulted in significantly higher blood loss of 361.11 ml compared to 118.48 ml for laminoplasty (*P* < 0.001) [41]. ACDF had a longer operative time of 187.78 min compared to 115.92 min for laminoplasty (*P* < 0.001) [41].

#### Recommendations

For CSM, the symptom severity at presentation reflected by the pre-operative JOA scores plays an important role in prognostication of postoperative recovery. If patients have a pre-operative JOA score ≥ 14, their recovery is near 100% regardless of the approach and for severe cases the recovery rate is still > 60% regardless of surgical approach. However, if the patient has pre-existing deformities such as cervical kyphosis, the anterior approach is preferred. In pre-existing kyphosis, posterior surgeries violate the posterior ligamentous complex and may exacerbate the deformity. The posterior approach is recommended for those with osteoporosis, chronic renal failure and smokers as there is high-risk of instrumentation failure for anterior approaches. Nevertheless, current evidence is still inconclusive and require further study. Studies which focused exclusively on multi-level CSM recommend posterior approaches as there are reported similar neurological outcomes and they are safer with less re-operations.

### Specifically OPLL

**Fig. 2:**
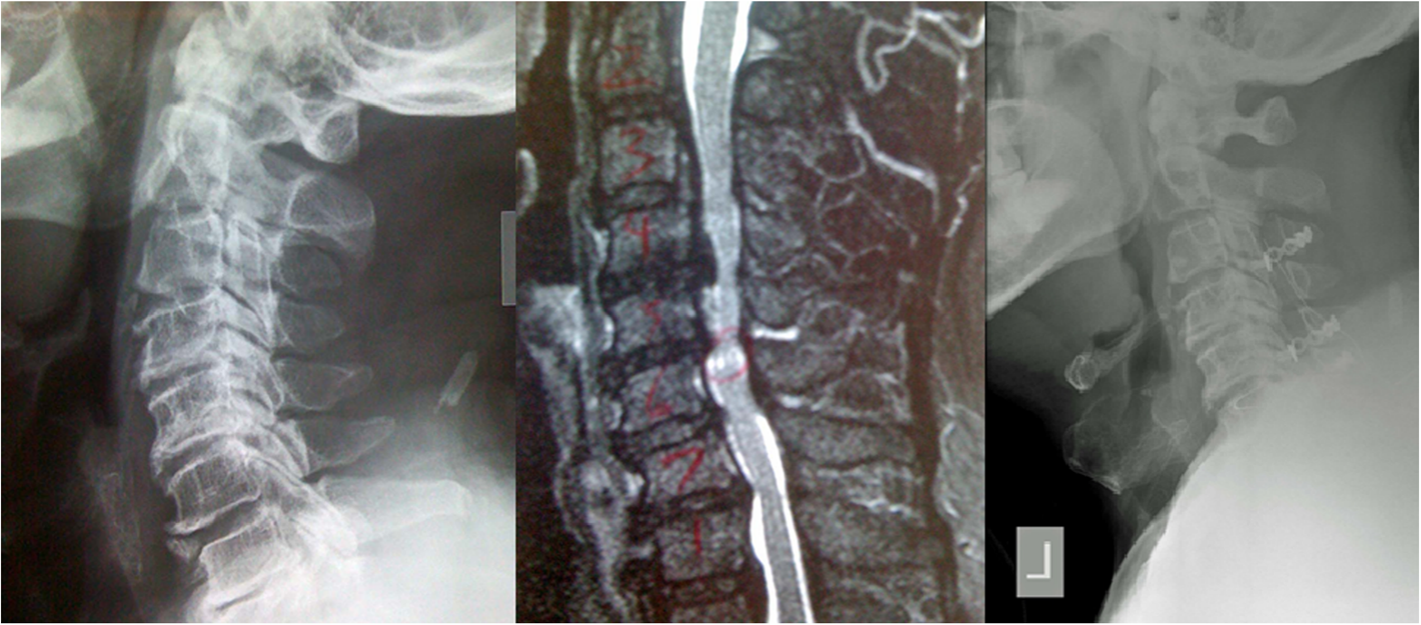
Pre-operative lateral x-ray of a patient with multi-level ossification of the posterior longitudinal ligament (left) and significant narrowing of spinal canal with myelomalacia at C5-C6 as seen on the T2-weighted MRI (middle). A laminoplasty fixed with miniplates was performed (right)

Cervical OPLL surgeries take up about 5% of cervical spine surgeries [43]. Anterior decompression often involves corpectomy followed by direct removal of the pathology or by floating technique, while the posterior approach involves indirect decompression via laminoplasty (Fig. 2) or laminectomy with fusion [44]. Laminectomy with fusion has been shown to maintain cervical lordosis better [45] and may slow the progression of OPLL [46]. However, there are far fewer comparative studies for cervical OPLL management as compared to CSM. The following will report only comparative studies for OPLL management.

#### Clinical outcome

**Fig. 3:**
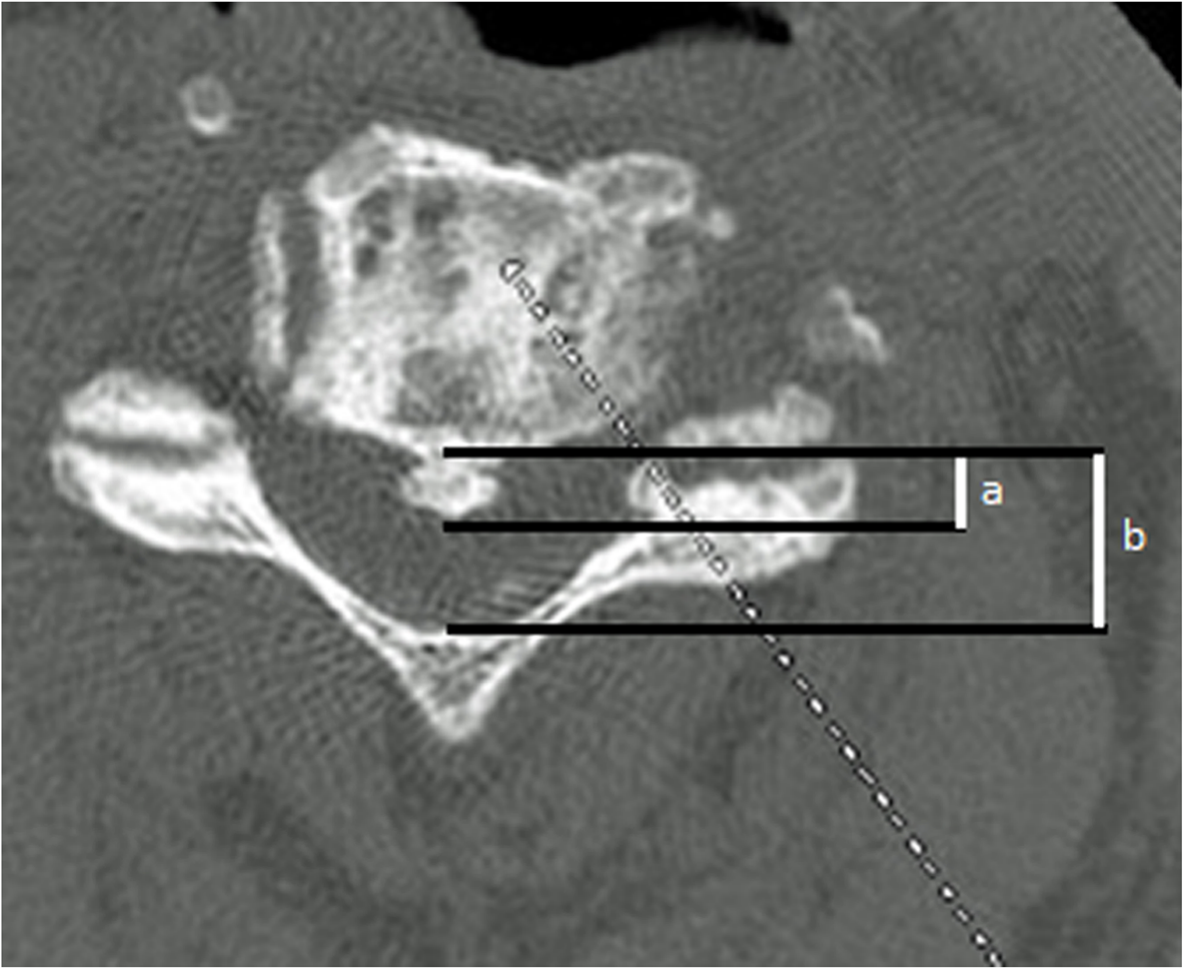
Spinal canal-occupying ratio calculated by dividing maximal ossification thickness (**a**) by the anteroposterior spinal canal diameter (**b**) on axial CT imaging

There is rather strong evidence suggesting that the canal-occupying ratio (Fig. 3) is a key determinant of surgical choice and ultimately clinical outcomes (Table 3). The canal-occupying ratio refers to the ratio of maximal ossification thickness to the anteroposterior spinal canal diameter on axial CT imaging [53]. A meta-analysis showed that anterior surgery provided better overall post-operative neural function than laminoplasty [18]. However, anterior surgery was preferable for patients with canal-occupying ratio > 50–60% since the post-operative JOA scores and recovery rates were significantly higher than the posterior approach in this group of patients (*P* < 0.01) [18]. Another study also reiterates that anterior surgery is generally preferred for those with large volume OPLL where indirect compression posteriorly may be insufficient [1]. However, laminoplasty is preferable for patients with canal-occupying ratio < 50–60%. In these cases, the post-operative JOA scores and recovery rates were similar for both anterior and posterior approaches but the anterior approach bears significantly greater surgical risks [18]. Iwasaki et al [47, 48] conducted a two-part study to compare the limitations of both the anterior and posterior approach for cervical OPLL. The first study was a retrospective analysis of 66 patients undergoing laminoplasty [47]. Those with spinal canal occupancy > 60% had mean recovery rate of only 14% (*P* < 0.03) at final follow-up while < 60% occupancy had recovery rate of 58% at final follow-up [47]. Hill-shaped lesions also showed poorer outcomes [47]. The second study looked at 27 patients who underwent anterior decompression [48]. The neurologic outcome was excellent or good in 56% of patients, fair in 37%, and poor in 7%. In the second part of the study with laminoplasty, excellent or good outcomes occurred in 65%, fair outcomes in 15% and poor outcomes in 20% [48]. The excellent or good outcome proportions of the anterior approach were similar to the posterior approach in the first study but the anterior approach had fewer poor outcomes [48].

**Table 3:** Comparison for OPLL

Reference	Type of study	No. of patients	Parameters measured	Key findings
Feng et al. (ref # [18])	Meta-analysis and systematic review	1050	Pre-operative and post-operative JOA scores with canal	Canal-occupying ratio < 50–60% • no significant difference between anterior and posterior post-op JOA scores Canal-occupying ratio > 50–60% • anterior approach preferred due to significantly higher post-op JOA score (*P* < 0.05)
Ma et al. 2018 (ref # [45])	Meta-analysis and systematic review	292	JOA score	No significant difference in the pre-operative and post-operative JOA scores in laminectomy and fusion vs laminoplasty
Lee et al. 2018 (ref # [46])	Retrospective series	83	Volume of OPLL using CT scans	Laminoplasty resulted in a mean annual growth rate of OPLL of about seven times of those who received laminectomy and fusion Laminoplasty provided better mobility than laminectomy and fusion
Iwasaki et al. 2007 (ref # [47])	Retrospective clinical study	66	mJOA and recovery rate after laminoplasty	Those with spinal canal occupancy < 60% had a significantly better recovery rate after laminoplasty than those with spinal canal occupancy > 60%
Iwasaki et al. 2007 (ref #[48])	Retrospective clinical study	27	mJOA and recovery after anterior decompression vs laminoplasty	Excellent or good outcome proportions of the anterior approach were similar to the posterior approach Anterior approach had fewer poor outcomes
Fujiyoshi et al. 2008 (ref # [49])	Non-randomized clinical trial	27	JOA scores before and one year after surgery after posterior decompression and mean recovery rate	K-line (−) patients are not suitable for laminoplasty due to posterior shift of the spinal cord K-line (+) patients are more suitable for posterior approach
Chen et al. 2011 (ref # [50])	Retrospective clinical study	75	JOA score	ACCF is superior to laminoplasty for multi-level OPLL ACDF vs laminectomy showed no significant difference in post-op JOA scores
Nayak et al. 2018 (ref # [51])	Meta-analysis	3963	5-year QALY	Laminoplasty had the highest 5-year QALYs gained compared to laminectomy and anterior approaches
Sun et al. 2018 (ref # [52])	Non-randomised clinical study	24	JOA scores K-line status	OPLL > 6 mm • K-line (−) had a better outcome than the K-line (+) group after anterior decompression OPLL < 6 mm • No difference in clinical outcomes of after anterior decompression

*ACCF* Anterior cervical corpectomy and fusion, *ACDF* Anterior cervical discectomy and fusion, *mJOA* Modified Japanese Orthopaedic Association, *OPLL* Ossification of posterior longitudinal ligament, *QALY* Quality-adjusted life years

**Fig. 4:**
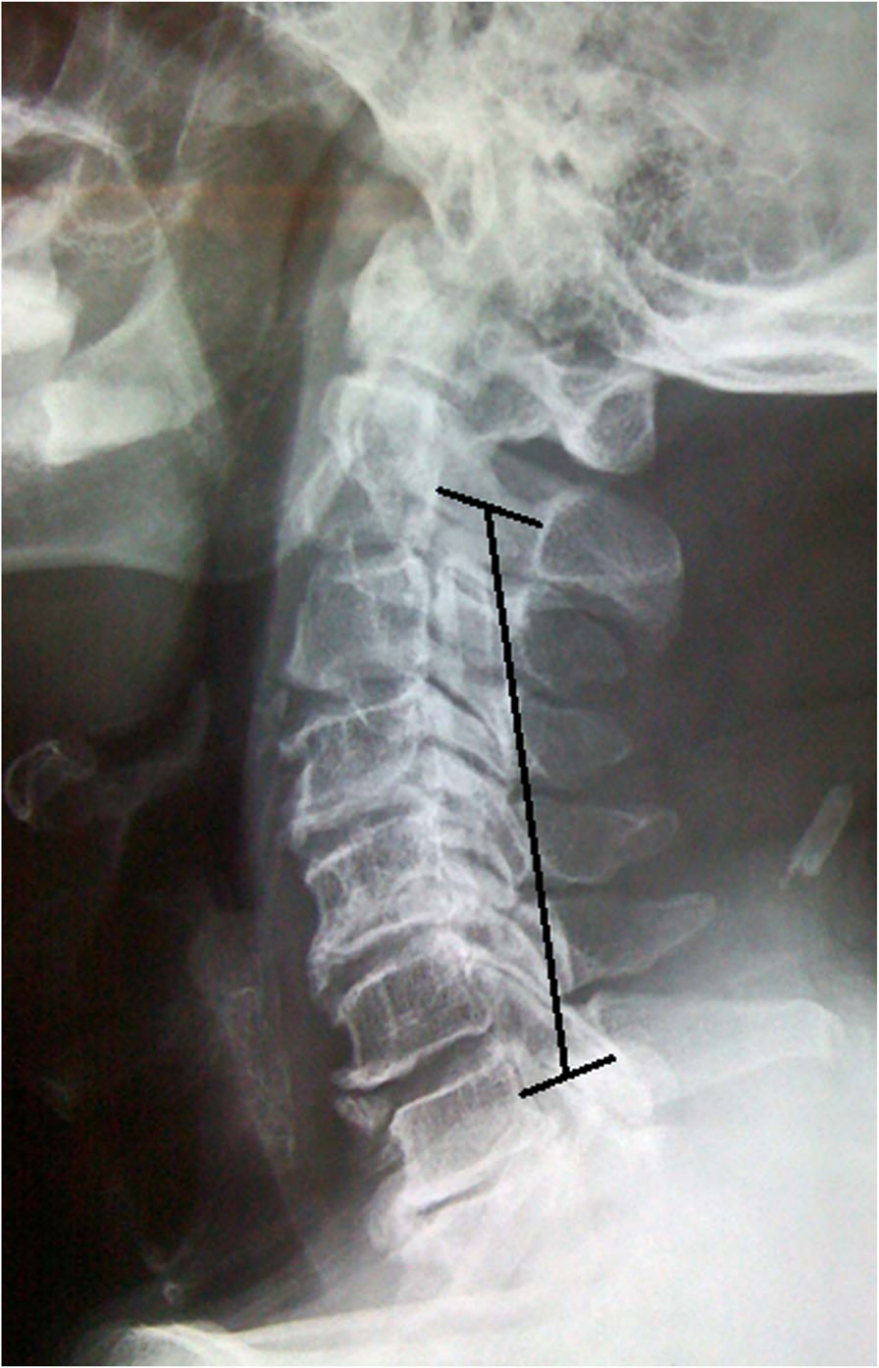
Lateral x-ray of a patient with an ossification of the posterior longitudinal ligament. The black line demonstrates the K-line drawn by linking the mid-point of anteroposterior canal diameter at C2 and C7

The K-line is an important consideration when deciding surgical management for OPLL. The K-line refers to a virtual line between the mid-point of anteroposterior canal diameter of C2 and C7 which assesses cervical spine alignment (Fig. 4) [54]. OPLL cases can be divided into K-line (+) group where the OPLL does not exceed the K-line and K-line (−) group which refers to OPLL beyond the K-line [49]. The posterior approach in K-line (−) patients, have shown to have insufficient spinal cord decompression and worsened neurological outcome thus the anterior approach is preferred in these cases [55]. A study of those with OPLL who underwent posterior decompression found that the mean recovery rate was 13.9% in the K-line (−) group and 66% in the K-line (+) group (*P* < 0.01) [49]. The authors ultimately concluded that for K-line (−), laminoplasty was not suitable since the posterior shift of the spinal cord is likely to be inadequate and recommended the anterior approach instead [49, 56]. However this study failed to address the issue of canal-occupying ratio which may influence the outcomes of surgery. There are no studies which consider the size of OPLL and K-line simultaneously when comparing the anterior and posterior approaches highlighting an important gap in our understanding.

A study which studied the surgical approach for severe multi-level OPLL found that ACCF was superior to laminoplasty due to significantly better post-operative JOA scores (*P* < 0.01) [50]. The same was not apparent for ACDF as there were no differences in the post-operative JOA scores when compared to laminectomy [50]. However, it is worth noting the surgical difficulties in attempting to remove a large OPLL anteriorly followed by reconstruction of the spine [50]. In contrast, another study which looked at the 5-year quality-adjusted life years (QALY) to compare different surgical approaches concluded that laminoplasty resulted in the highest 5-year QALYs gained, compared to laminectomy and anterior approaches (*P* < 0.001) [51]. They also concluded that since the anterior approach has more complications, laminoplasty is the recommended approach [51]. A study which looked at patients with multi-level OPLL who underwent anterior decompression, found that when the OPLL was thin (≤6 mm), there was no difference in clinical outcomes regardless of K-line status. However, for OPLL > 6 mm, the K-line (−) had a better outcome than the K-line (+) group [52]. A survey study amongst spine surgeons in the Asia-Pacific region concludes that anterior decompression was recommended for one- or two-level disease, LP was preferred for two- to four-level disease and LP was preferred for multi-level OPLL [26]. This suggests that for multi-level OPLL, considering both the size and K-line is important for predicting the outcomes of patients. Comparably, laminectomy and fusion is more popular in North America and Europe [37]. Nevertheless, decision-making is likely influenced by surgical experience [57]. Most surgeons determined their approached based on the location of the compression. High degree of anterior compression or cervical kyphosis warranted an anterior approach. Most Asian surgeons generally preferred posterior approaches. The finding of focal OPLL showed marked variance. The median North American surgeon may favour posterior approach while others preferred anterior approaches. In contrast, posterior approaches are preferred for cases with congenital canal stenosis or multilevel OPLL.

One study which compared laminoplasty versus laminectomy and fusion for patients with multi-level OPLL showed that laminectomy and fusion was superior in maintaining cervical lordosis compared to laminoplasty in patients with OPLL [58]. There was also no significant difference in pre-operative and post-operative JOA scores of patients in either approach so both methods could achieve similar clinical improvement [58]. There was no significant difference in the rate of C5 palsy and axial pain with either approach but laminectomy and fusion had more blood loss (*P* < 0.03) [58]. Another study showed that laminectomy and fusion was able to significantly reduce the OPLL growth rate compared to laminoplasty [46]. OPLL volumes were assessed using CT scan pre and post-operatively and showed that patients who underwent laminoplasty had a mean annual growth rate of OPLL of about seven times that of those who underwent laminectomy and fusion [46]. Although laminoplasty provides better mobility, there is risk of earlier recurrence of symptoms and reoperation. A survey study amongst spine surgeons in the Asia-Pacific region and the pooled recommendations included anterior decompression for one- or two-level disease and laminoplasty for multi-level disease [26]. Whether laminoplasty or laminectomy and fusion is superior requires further evidence.

#### Complications

An important complication of the anterior approach for OPLL is dural tear with the risk ranging from 4.3 to 32% [59–61]. In comparison, the risk of dural tears in the posterior approach is only 0.5–3%. The anterior approach had a notably higher complication rate with 15% developing graft related complications, 7% developing neurological deterioration and 26% ultimately needing additional surgical interventions [48]. As for the posterior approach the complication rate was lower with 9% transient upper extremity paresis, 8% persistent neuropathic arm pain and 1% reoperation rate [47]. Generally speaking, the complication rate for the anterior approach is significantly higher than the posterior approach for the OPLL surgery.

#### Recommendations

For OPLL, choosing the approach depends mainly on the K-line and canal-occupying ratio. For K-line (−) patients regardless of canal occupying ratio, anterior approach should be adopted due poor surgical outcomes of the posterior approach. For K-line (+) patients with canal-occupying ratio > 60%, the anterior approach is preferred (Table 4). For K-line (+) with canal-occupying ratio < 50–60%, the posterior approach is preferred. These findings are consistent with other studies which suggested that the anterior approach is preferred for a spinal canal-occupying ratio > 60% compared to the posterior approach due to better recovery rates [48, 62, 63]. Studies focusing on multi-level OPLL again confirmed the importance of assessing size of OPLL and the K-line status. For large multi-level OPLL which is K-line (+) with small OPLL (< 6 mm), the posterior approach specifically laminoplasty is the preferred surgical approach. When choosing the posterior approach for OPLL it is important to recognise that both laminoplasty and laminectomy and fusion have their own respective advantages and disadvantages. It is important to recognise laminoplasty provides better post-op mobility which may be more important in younger patients and those who are working [58]. However, there is a risk for OPLL to increase in size in motion-preservation surgery.

**Table 4:** Recommendations

	Pathology specific indications
**Anterior approach**	DCM:
	• Pre-existing cervical kyphosis
	OPLL:
	• K-line (−) patients regardless of canal-occupying ratio
	• K-line (+) and canal-occupying ratio > 60%
**Posterior approach**	DCM:
	• Severe osteoporosis, renal failure, smokers
	• Multi-level pathology
	OPLL:
	• K-line (+) and canal-occupying ratio < 50–60%
	• Multi-level pathology

*DCM* Degenerative cervical myelopathy, *OPLL* Ossification of the posterior longitudinal ligament

## Discussion and future directions

Current evidence consistently showed that ACDF had significantly higher intra-operative and post-operative risks regardless of the pathology operated on [18, 37, 38, 40, 48]. However, this finding does seem to be pathology specific as the anterior and posterior approaches have similar complication rate when adjusting for severity in CSM cases. Thus, it is important to note the pre-operative JOA and NDI score when planning surgery for CSM.

It is worth noting that there are no studies which look at K-line and canal-occupying ratio together when deciding between approaches. Hence, this is certainly a future research direction. It is important to note though that certain conditions may preclude the use of an anterior or posterior approach. For example in patients with multi-level OPLL, most surgeons may not consider the anterior approach at all. For CSM, the results remain rather controversial as there does not seem to be a significant difference in the long-term outcome between the two approaches but only suggestion of better immediate post-operative neurological recovery. This would be an important point in counselling the patient as they should be told that anterior approach may lead to more rapid symptomatic relief though the final outcome may be similar. The evidence however is not as clear for multi-level OPLL due to the lack of comparative studies.

The debate between ACDF and laminoplasty or laminectomy and fusion is likely to continue as we have yet to have high quality studies showing which approach is more effective. Ultimately, management options will need to be individualised. Given that there are numerous surgical techniques available, it is not surprising to find numerous factors that should be considered when planning for surgery. These include the type and location of the pathology, patient co-morbidities and the severity at presentation. The majority of studies also may have combined various causes of myelopathy in their analyses. Despite CSM being the most common cause of cervical myelopathy, acute disc herniations and OPLL may confound the results of the study. The disease type and location should be separated for study. One example is a large-scale RCT which looked at 757 patients and used preoperative MRI to characterise the nature of the compression. Propensity scores which represent the estimated probability of undergoing anterior decompression were calculated by considering pre-op MRI findings, JOA scores and demographic data [42]. Propensity score matched analysis have been shown to reduce confounding bias [64]. Modified JOA score was used to assess the post-operative outcome during the follow-up period of 2 years and showed no significant differences between approaches [42]. More studies of such detailed methodology is necessary.

Even though there are prospective clinical trials comparing different approaches, it is worth noting that many of these studies cannot be randomised since the results are not blinded and thus the interpretation of these results should be interpreted with caution. Regardless, we still need more large scale clinical trials to see if the data is reproducible. In addition, there are no studies comparing the different approaches in patients with osteoporosis. Cost-effectiveness is also an important consideration when deciding surgical management. A study comparing the cost of ACDF vs posterior cervical decompression and fusion demonstrated that posterior surgery had significantly higher hospital charges [65]. There is a new clinical trial (ClinicalTrials.gov identifier: NCT02076113) that has been completed recruitment but yet to be published addressing the differences in outcomes between anterior decompression and fusion and posterior decompression with laminoplasty or fusion. This study may shed light on the optimal surgical approach for patients with multi-level DCM.

## Conclusions

Deciding between ACDF and laminoplasty or laminectomy and fusion for DCM and OPLL remains controversial. Anterior approach is preferred for K-line (−) OPLL, K-line (+) with canal occupying ratio > 60% and DCM with pre-existing cervical kyphosis. Posterior approach is preferred for K-line (+) OPLL with canal-occupying ratio < 50–60%, and multi-level DCM. The anterior approach is associated with more complications regardless of pathology and thus needs to be weighed carefully when deciding between approaches.

## Data Availability

Data sharing is not applicable to this article as no datasets were generated or analysed during the current study.

## Abbreviations

ACDF: Anterior cervical discectomy with fusion
ACCF: Anterior cervical corpectomy with fusion
CSM: Cervical spondylotic myelopathy
DCM: Degenerative cervical myelopathy
JOA: Japanese Orthopaedic Association
NDI: Neck Disability Index
OPLL: Ossification of the posterior longitudinal ligament
QALY: Quality-adjusted life years
